# Evaluating oxidative stress targeting treatments in *in vitro* models of placental stress relevant to preeclampsia

**DOI:** 10.3389/fcell.2025.1539496

**Published:** 2025-02-28

**Authors:** Dinara Afrose, Matt D. Johansen, Valentina Nikolic, Natasa Karadzov Orlic, Zeljko Mikovic, Milan Stefanovic, Zoran Cakic, Philip M. Hansbro, Lana McClements

**Affiliations:** ^1^ School of Life Sciences, Faculty of Science, University of Technology Sydney, Sydney, NSW, Australia; ^2^ Centre for Inflammation, Centenary Institute and University of Technology Sydney, Sydney, NSW, Australia; ^3^ Department of Pharmacology with Toxicology, Faculty of Medicine, University of Nis, Nis, Serbia; ^4^ Department of Gynaecology and Obstetrics, Narodni Front, Belgrade, Serbia; ^5^ Faculty of Medicine, University of Belgrade, Belgrade, Serbia; ^6^ Department of Gynaecology and Obstetrics, Clinical Centre Nis, Nis, Serbia; ^7^ Department of Gynaecology and Obstetrics, Faculty of Medicine, University of Nis, Nis, Serbia; ^8^ Department of Gynaecology and Obstetrics, General Hospital of Leskovac, Leskovac, Serbia; ^9^ Institute for Biomedical Materials and Devices, Faculty of Science, University of Technology Sydney, Sydney, NSW, Australia

**Keywords:** oxidative stress, preeclampsia, trophoblast cells, placenta, resveratrol, pregnancy, aspirin, metformin

## Abstract

**Background:**

Preeclampsia is a complex pregnancy disorder characterized by the new onset of hypertension and organ dysfunction, often leading to significant maternal and fetal morbidity and mortality. Placental dysfunction is a hallmark feature of preeclampsia, which is often caused by inappropriate trophoblast cell function in association with oxidative stress, inflammation and/or pathological hypoxia. This study explores the role of oxidative stress in trophoblast cell-based models mimicking the preeclamptic placenta and evaluates potential therapeutic strategies targeting these mechanisms.

**Methods:**

Uric acid (UA) and malondialdehyde (MDA) concentrations were measured in human plasma from women with preeclampsia (n = 24) or normotensive controls (n = 14) using colorimetric assays. Custom-made first trimester trophoblast cell line, ACH-3P, was exposed to various preeclampsia-like stimuli including hypoxia mimetic (dimethyloxalylglycine or DMOG, 1 mM), inflammation (tumour necrosis factor or TNF-α, 10 ng/mL) or mitochondria dysfunction agent, (Rhodamine-6G or Rho-6G, 1 μg/mL), ± aspirin (0.5 mM), metformin (0.5 mM), AD-01 (100 nM) or resveratrol (15 µM), for 48 h. Following treatments, UA/MDA, proliferation (MTT), wound scratch and cytometric bead, assays, were performed.

**Results:**

Overall, MDA plasma concentration was increased in the preeclampsia group compared to healthy controls (p < 0.001) whereas UA showed a trend towards an increase (p = 0.06); when adjusted for differences in gestational age at blood sampling, MDA remained (p < 0.001) whereas UA became (p = 0.03) significantly correlated with preeclampsia. Our 2D first trimester trophoblast cell-based *in vitro* model of placental stress as observed in preeclampsia, mimicked the increase in UA concentration following treatment with DMOG (p < 0.0001), TNF-α (p < 0.05) or Rho-6G (p < 0.001) whereas MDA cell concentration increased only in the presence of DMOG (p < 0.0001) or Rho-6G (p < 0.001). Metformin was able to abrogate DMOG- (p < 0.01), Rho-6G- (p < 0.0001) or TNF-α- (p < 0.01) induced increase in UA, or DMOG- (p < 0.0001) or TNF-α- (p < 0.05)induced increase in MDA. AD-01 abrogated UA or MDA increase in the presence of TNF-α (p < 0.001) or Rho-6G (p < 0.001)/DMOG (p < 0.0001), respectively. The preeclampsia-like stimuli also mimicked adverse impact on trophoblast cell proliferation, migration and inflammation, most of which were restored with either aspirin, metformin, resveratrol, or AD-01 (p < 0.05).

**Conclusion:**

Our 2D *in vitro* models recapitulate the response of the first trimester trophoblast cells to preeclampsia-like stresses, modelling inappropriate placental development, and demonstrate therapeutic potential of repurposed treatments.

## Graphical Abstract

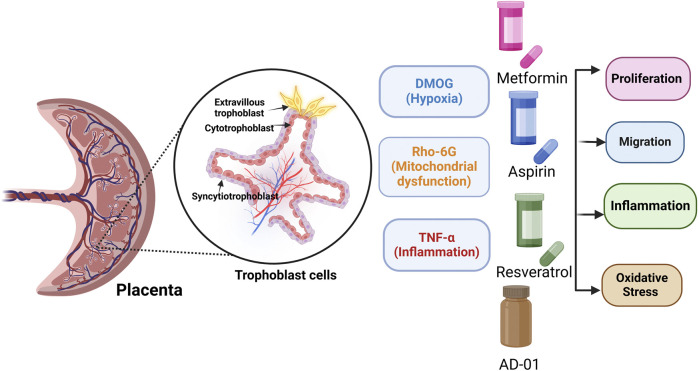

## Background

Preeclampsia is a pregnancy-related multiorgan disorder characterized by high blood pressure (BP > 140/90), proteinuria or organ dysfunction including placenta and can be classified into an early-onset (diagnosed <34-week gestation), late-onset (diagnosed ≥34 weeks) or *postpartum* phenotype (Dimitriadis et al., 2023) (Brownfoot and Rolnik, 2024) (Steegers et al., 2010). However, the evolving understanding of preeclampsia as a heterogeneous hypertensive disorder of pregnancy led to the ACOG’s hypertension 2019 task force revising the definition of preeclampsia to include the presence of other features including elevated liver enzymes, low platelet count, headache, with or without proteinuria (ACOG, 2019). Globally, preeclampsia is the leading cause of morbidity and mortality among pregnant women and their offspring. It can lead to severe pregnancy complications including eclampsia, Haemolysis, Elevated Liver Enzymes and Low Platelets (HELLP) syndrome, preterm birth, and even death if it is not detected and managed in a timely manner. Long-term, both the mothers and their offspring are at an increased risk of developing cardiovascular and metabolic disorders, later in life (Rana et al., 2019; Sibai et al., 2005; Matthys et al., 2004; Tranquilli et al., 2014; Lopez-Jaramillo et al., 2018). Research indicates that maternal mortality related to preeclampsia is disproportionately higher in low-income and lower socioeconomic countries. Currently, preeclampsia has limited monitoring options, with the only definitive treatment being the delivery of the placenta and baby, often preterm and associated with significant complications (Dimitriadis et al., 2023; Ives et al., 2020). Although the exact etiology of preeclampsia remains unclear, it is often associated with placental dysfunction or cardiovascular maladaptation, leading to excessive oxidative stress, inflammation, endothelial dysfunction, and an antiangiogenic environment (Jung et al., 2022; Bokuda and Ichihara, 2023).

In the early stages of physiological pregnancy, trophoblast cells invade the decidualized endometrial lining of the uterus and remodel the spiral uterine arteries (SUA), ensuring a stable connection between the placenta and maternal circulation. An invasive subtype of extravillous trophoblasts (EVTs) invades the SUA within the decidua in a tightly regulated process, replacing endothelial and muscle layers, thereby reducing vessel resistance and ensuring uninterrupted blood flow to the fetus (Ridder et al., 2019; Pollheimer et al., 2018). However, in some forms of preeclampsia, the extent and depth of remodeling are less extensive than in a normal pregnancy. Following compromised placentation, impaired SUA remodeling can trigger a cascade of events (Burton et al., 2019). For instance, placental ischemia and inflammation lead to the upregulation of anti-angiogenic proteins, including soluble fms-like tyrosine kinase-1 (sFlt-1), soluble endoglin (sEng) and FK506 binding protein-like (FKBPL), (Todd et al., 2021; Agarwal and Karumanchi, 2011), as well as oxidative stress biomarkers, including uric acid (UA) and malondialdehyde (MDA) (Afrose et al., 2022; Sudjai and Satho, 2022; Khaliq et al., 2018; Yoneyama et al., 2002; Rani et al., 2010). The angiogenic imbalance in association with mitochondrial dysfunction and oxidative stress can ultimately lead to organ damage. Preeclampsia is associated with an exacerbated inflammatory response that may lead to a release of pro-inflammatory cytokines, including TNF-α, interleukin-6 (IL-6), interleukin-8 (IL-8), and interleukin-1 beta (IL-1β). A dysregulated immune response observed in preeclampsia leads to endothelial activation, oxidative stress, and the release of factors that perpetuate the inflammatory reaction (Maynard and Karumanchi, 2011; Martínez-Varea et al., 2014; Geldenhuys et al., 2018)

Even though there is no definitive treatment for preeclampsia, several preventative measures are utilized including lifestyle modifications, exercise, and adequate rest (Bezerra Maia e Holanda Moura S et al., 2012; Davenport et al., 2018). Low-dose aspirin (100–162 mg/day) has been established as an effective prophylactic or preventative treatment for preterm preeclampsia (delivery prior to 37 weeks of gestation), when prescribed prior to 16 weeks of gestation (Rolnik et al., 2017; Bujold et al., 2010; Duley et al., 2007). Several newly emerging treatments for preeclampsia are currently being investigated, through repurposing, including metformin and resveratrol. Metformin is a hypoglycaemic agent with pleiotropic properties, which, in a recent randomised controlled trial in South Africa, showed the ability to extend gestation period in early-onset preeclampsia by an average of 7 days (Alqudah et al., 2018; Cluver et al., 2021; Nafisa et al., 2018). Meta-analyses of studies on high-risk, insulin-resistant women demonstrated that metformin use before or during pregnancy is associated with reduced gestational weight gain and a lower risk of preeclampsia compared to insulin therapy alone (Alqudah et al., 2018). Metformin can improve endothelial function and vasculature while reducing the secretion of sFlt-1 and sEng from human placental tissues, potentially through inhibiting the mitochondrial electron transport chain, further supporting its therapeutic potential in preeclampsia (Brownfoot et al., 2016). Resveratrol is a naturally occurring compound, found in grape skin with anti-inflammatory and antioxidatant properties, and a therapeutic potential in cancer, inflammatory lesions, diabetes mellitus, and cardiovascular disease (Zou et al., 2014) (Lacerda et al., 2023; Singh et al., 2019). A recent study demonstrated that resveratrol improves metabolic health in pregnant individuals and their offspring, and was deemed safe in pregnancy at certain doses (Ramli et al., 2023). Resveratrol appears to enhance the invasive capacity of human trophoblasts by promoting the epithelial-mesenchymal transition (EMT) process, potentially through targeting the Wnt/β-catenin signalling pathway, therefore suggesting that it could be a promising treatment for prevention of preeclampsia (Zou et al., 2019). FKBPL has emerged as a new predictive and diagnostic biomarker and a therapeutic target of preeclampsia (Masoumeh Ghorbanpour et al., 2023; Ghorbanpour et al., 2023; McNally et al., 2021; Todd et al., 2021). FKBPL-based therapeutic peptide mimetic, AD-01 (preclinical peptide candidate), has showed a potent anti-angiogenic and anti-cancer stem cell effects in cancer via CD44 and DLL4 (Yakkundi et al., 2013; McClements et al., 2013; Annett et al., 2020). More recently, AD-01 has also demonstrated an anti-inflammatory utility through the inhibition of NF-kB signalling in association with improved vascular dysfunction (Annett et al., 2021). In cardiovascular disease context, AD-01 was able to restore angiotensin-II-induced cardiac hypertrophy via negative regulation of FKBPL, which could make it useful for preeclampsia treatment although its safety in pregnancy is unknown (Chhor et al., 2023).

In this study, we aimed to design a range of the first trimester trophoblast cell-based *in vitro* models emulating placental stresses preceding preeclampsia to elucidate the impact of individual pathogenic mechanisms and potential therapeutics for preeclampsia prevention. We showed that in our low-cost, representative and reproducible 2D *in vitro* models, we can mimic oxidative stress (increased UA and MDA), impaired trophoblast proliferation and migration, and inflammation, typical for inappropriate placentation leading to preeclampsia. Our comprehensive evaluation of potential treatments shows, for the first time, that through repurposing metformin, resveratrol and AD-01, we can restore the negative impact of preeclampsia-like stresses on trophoblast function, oxidative stress and inflammation.

## Methods

### Human sample collection

Human plasma samples were collected as part of a multicentre study including three hospitals in Serbia. A total of 38 blood samples were used from participants with preeclampsia (n = 24) or healthy controls (n = 14) of matched age, body mass index (BMI) and blood glucose levels. The samples were collected prior to delivery as previously described (Ghorbanpour et al., 2023). Plasma was isolated from blood samples collected using ethylenediaminetetraacetic acid (EDTA) tubes, by centrifugation at 3,000 g for 10 min at 4°C. To preserve the samples, plasma-containing tubes were stored at −80°C. Preeclampsia was defined in accordance with the ACOG 2019 guidelines, ensuring robust sample characterisation (ACOG, 2019). Clinical characteristics of maternal age, gestational age at sampling/delivery, maternal BMI, systolic blood pressure (sBP), diastolic blood pressure (dBP), blood glucose and concentrations of UA and MDA are presented in Table 1.

**TABLE 1:** Clinical characteristics of normotensive pregnancies and pregnancies with established preeclampsia.

	Control (n = 14)	Preeclampsia (n = 24)	P value
Age (years)	31.14 ± 4.17	33.83 ± 6.58	0.181
Gestational age at delivery (weeks)	39.49 ± 0.82	32.88 ± 3.59	**<0.0001**
BMI (kg/m^2^)	25.67 ± 5.72	27.38 ± 4.43	0.313
sBP (mmHg)	115 ± 6.50	155.1 ± 24.13	**<0.0001**
dBP (mmHg)	73.57 ± 7.45	102.8 ± 9.88	**<0.0001**
Blood Glucose (mmol/L)	4.43 ± 0.83	4.38 ± 0.88	0.871
UA (nmol/μL)	33.55 ± 7.87	40.13 ± 10.88	0.06
MDA (μmol/mL)	6.72 ± 0.86	12.27 ± 1.28	**<0.0001**

All clinical characteristics are given as mean ± SD., Bold indicates statistical significance (p < 0.0001). Key: BMI, body mass index; sBP, systolic blood pressure; dBP, diastolic blood pressure; UA, uric acid; MDA, malondialdehyde.

### Cell culture

Professor Gernot Desoye (Graz Medical University, Austria) generously donated the ACH-3Ps first trimester trophoblast cell line, which was established in 2007 (Hiden et al., 2007). ACH-3Ps were immortalized by fusing primary first trimester trophoblast cells from a 12-week gestation placenta with choriocarcinoma cell line, AC1-1 (Hiden et al., 2007). Ham’s F12 nutrient mix (Gibco; Thermo Fisher Scientific, cat. 11765062) was used for the ACH-3P cell culture, supplemented with 10% fetal bovine serum (FBS; Gibco; Thermo Fisher Scientific, cat. 10099141) and 1% penicillin-streptomycin (P/S; Gibco; Thermo Fisher Scientific, cat. 15140-122). A selection medium containing azaserine (5.7 M, Sigma-Aldrich) and hypoxanthine (100 M, Sigma-Aldrich) was applied to cells every two to five cell passages. A humidified atmosphere was used to incubate the cells at 37°C, 5% CO_2_ and the cultures were routinely tested for the presence of *Mycoplasma*. The cells were dissociated with Accutase (Sigma-Aldrich, cat. A6964) and experiments were conducted at passages P15-25.

### Cell stimuli and treatments

ACH-3Ps were seeded at various seeding densities in different size well plates for various sets of experiments (MTT assay, wound-scratch assay, cytometric bead assay (CBA) assay, UA assay and MDA assay) and incubated in a humidified environment at 37°C and 5% CO_2_. After attaching, cells were serum starved overnight by using serum reduced medium with Ham’s F12 containing 1% FBS, 1% P/S prior to treatments. The following day, cells were treated with stimuli as previously described including 1 mM DMOG (Sigma-Aldrich, United States, cat. D3695) (Nevo et al., 2006; Zippusch et al., 2021) to mimic hypoxic, 10 ng/mL TNF-α (Sigma-Aldrich, United States, cat. T6674) (Brownfoot et al., 2020; Wang et al., 2020) to mimic inflammatory condition, or 1 μg/mL Rho-6G (Sigma-Aldrich, United States, cat. R4127) (Dutra Silva et al., 2021; Gear, 1974) to mimic mitochondrial dysfunction ± metformin (Cluver et al., 2021; Nafisa et al., 2018; Han et al., 2015) (0.5 mM, Sigma-Aldrich, United States, cat. PHR1084), or ± AD-01 (McClements et al., 2013; McClements et al., 2019) (100 nM, MedChemExpress, United Kingdom, cat. HY-P2284), or ± aspirin (Bujold et al., 2010; Panagodage et al., 2016) (0.5 mM, Sigma-Aldrich, United States, cat. PHR1003), or ± resveratrol (Viana-Mattioli et al., 2020; Cluver et al., 2018; Ding et al., 2017) (15 μM, Sigma-Aldrich, United States, cat. R5010), for 48 h with untreated cells being used as a control. Drug dosages were optimized initially and chosen according to the concentrations reflective of human doses for aspirin and metformin following absorption, metabolism, distribution or plasma levels (Gong et al., 2012; Angiolillo et al., 2022).

### ACH-3P cell lysates extraction for UA and MDA assays

ACH-3Ps cells were seeded to achieve a total of 3 × 10^6^ cells per condition and incubated in a humidified environment at 37°C and 5% CO_2_ for 6 h. Cells were then starved overnight in serum reduced Ham’s F12 media containing 1% FBS, 1% P/S prior to adding the treatments. Following the addition of the treatments, cells were washed with cold Phosphate Buffer Saline (PBS) (2 mL) and homogenized with 100 µL UA assay buffer (Abcam, Australia, cat. ab65344). Samples were centrifuged at 14,000 rpm for 2 min at 4°C using a cold microcentrifuge to remove any insoluble material. The supernatant was collected and stored at - 80°C for downstream analysis. For the MDA assay, the same procedure was followed except that before extracting MDA, MDA lysis solution (Abcam, Australia, cat. ab118970) was prepared by mixing 300 µL of MDA lysis buffer with 3 μL Butylated Hydroxytoluene (BHT) (1:100). The purpose of using BHT was to stop further sample peroxidation during sample processing. Cells were homogenised properly until the shiny ring containing the nuclei was removed. Samples were centrifuged at 13,000 rpm for 10 min at 4°C to remove any insoluble material. The supernatant was collected and stored at - 80°C for downstream analysis.

### UA and MDA assay

Plasma from women with normotensive pregnancies or preeclampsia and cell lysates from treated ACH-3Ps were used to determine the concentration of UA and MDA. UA assay kit (Abcam, Australia, cat. ab65344) and MDA assay kit (Abcam, Australia, cat. ab118970) were used according to the manufacturer’s instructions. Optical density was measured for both analytes (UA and MDA) in plasma and ACH-3Ps cell lysate, samples using a Spark 10 M plate reader (Tecan, Switzerland) at optical density (OD) of 570 nm. The four-parameter logistic (4 PL) curve regression model was used to determine concentration values of each sample from the sigmoidal standard curve for both assays (UA and MDA).

### MTT assay

An MTT assay was performed using Thiazolyl Blue Tetrazolium Bromide dye (Sigma Aldrich) according to the manufacturer’s instructions. MTT assay is a well-established, cost-effective, and widely used method for assessing cell proliferation and viability. The ACH-3P cells were seeded at a concentration of 15,000 cells/well in triplicate wells of a 96-well plate. Cells were serum starved in serum reduced medium (Ham’s F12 containing 1% FBS, 1% P/S) overnight. Cells were then incubated in 210 μL medium spiked with PBS or hypoxic stimuli, 1 mM DMOG, mitochondrial dysfunctional stimuli 1 μg/mL Rho-6G or TNF-α (10 ng/ml) ± metformin (0.5 mM) or aspirin (0.5 mM) ± AD-01 (100 nM) ± resveratrol (15 μM) for 24 h, 48 h or 72 h prior to the addition of MTT dye. Next, 20 µL of the MTT reagent was added to each well, and the plates were incubated for 2 h at 37°C. The MTT reagent was then removed, and the resulting formazan crystals were solubilized in 200 µL of dimethylsulfoxide (DMSO; Sigma-Aldrich). The plates were shaken for 10 min to ensure complete solubilization of the crystals. Absorbance was measured at 565 nm using the Tecan Infinite M Plex plate reader (Tecan Life Sciences) and a well containing DMSO was used as a blank.

### Wound scratch assay

ACH-3Ps cells were seeded in 24-well plates at a concentration of 400,000 cells per well. Cells were incubated in serum reduced medium as described above overnight prior to the addition of treatments. The following day, a single vertical scratch using a P200 tip from the top to the bottom of each well was applied, before washing the cells twice with 200 µL PBS and replacing the starvation media with Ham’s F12 containing 10% FBS, 1% P/S. Next, treatments were added to include PBS as a control group or hypoxic stimuli (1 mM DMOG) ± metformin (0.5 mM or 1.0 mM or 5.0 mM) or ± aspirin (0.1 mM or 0.5 mM) or ± AD-01 (100 nM). Images of each well were taken using the Evos FL Live Cell imaging system (BioScience) and two × 10 objective images were acquired from each well at 0 h, 24 h and 48 h respectively. A wound area was measured, and a percentage of wound closure was calculated using ImageJ. Experiments were conducted in duplicates, and results are expressed as the mean percentage of wound closure ± standard error.

### Cytometric bead assay (CBA)

ACH-3Ps cells were seeded onto 24-well plates at the density of 500,000 cells/well before being serum-arrested in their respective medium containing 1% FBS and 1% P/S, overnight. The following day, cells were treated with hypoxic stimuli, 1 mM DMOG, as described above or mitochondrial dysfunctional stimuli, 1 μg/mL Rho-6G, ± metformin (0.5 mM) or ± AD-01 (100 nM), for 48 h with untreated cells being used as a control. The supernatant was collected and stored for measuring inflammatory cytokines (Human IFN-α, IL-1β, IL-6, IL-8, IL-10) at - 80°C.

Inflammatory cytokines from ACH-3Ps cells supernatant were quantified using the CBA according to the manufacturer’s instructions (Becton Dickinson, United States). A standard curve was generated using the standards provided for each analyte. In a 96-well plate, 10 µL of each sample was added to each well, followed by incubation with 1 µL of capture beads for each analyte (1 h, room temperature, in the dark). For each analyte captured, 1 µL of detection bead was added to each well, followed by room temperature (RT) incubation (2 h, in the dark). A solution of 8% paraformaldehyde was then used to fix the samples overnight (4% final solution). In order to examine the samples, a BD LSR Fortessa equipped with a High-Throughput Sampler (HTS) plate reader was used. FCAP Array software was used to facilitate the analysis of CBA Human Inflammatory Cytokines Kit data of standards and samples.

### Statistical analysis

The results of human sample quantifications were presented as mean ± SD, whereas the results of quantitative *in vitro* experiments were presented as mean ± SEM. Normality testing was performed using a Shapiro–Wilk test followed by two-tailed unpaired t-test, or one-way ANOVA with post-hoc multiple comparison tests. For non-normally distributed data, Mann–Whitney or Kruskal–Wallis were used. Statistical analysis was performed using Graph-Pad Prism (version 9.4.0 software, United States) and p value <0.05 was considered statistically significant. An unpaired t-test was used to determine differences between normotensive pregnancy and preeclampsia groups. SPSS software (IBM SPSS Statistics, 29.0.2.0, United States) was used to perform correlations between preeclampsia and UA or MDA plasma concentration using Pearson’s correlation and partial correlation controlling for differences in gestational age (GA).

## Results

### UA and MDA are increased in preeclampsia plasma samples and in the *in vitro* models of preeclamptic placenta

**FIGURE 1:**
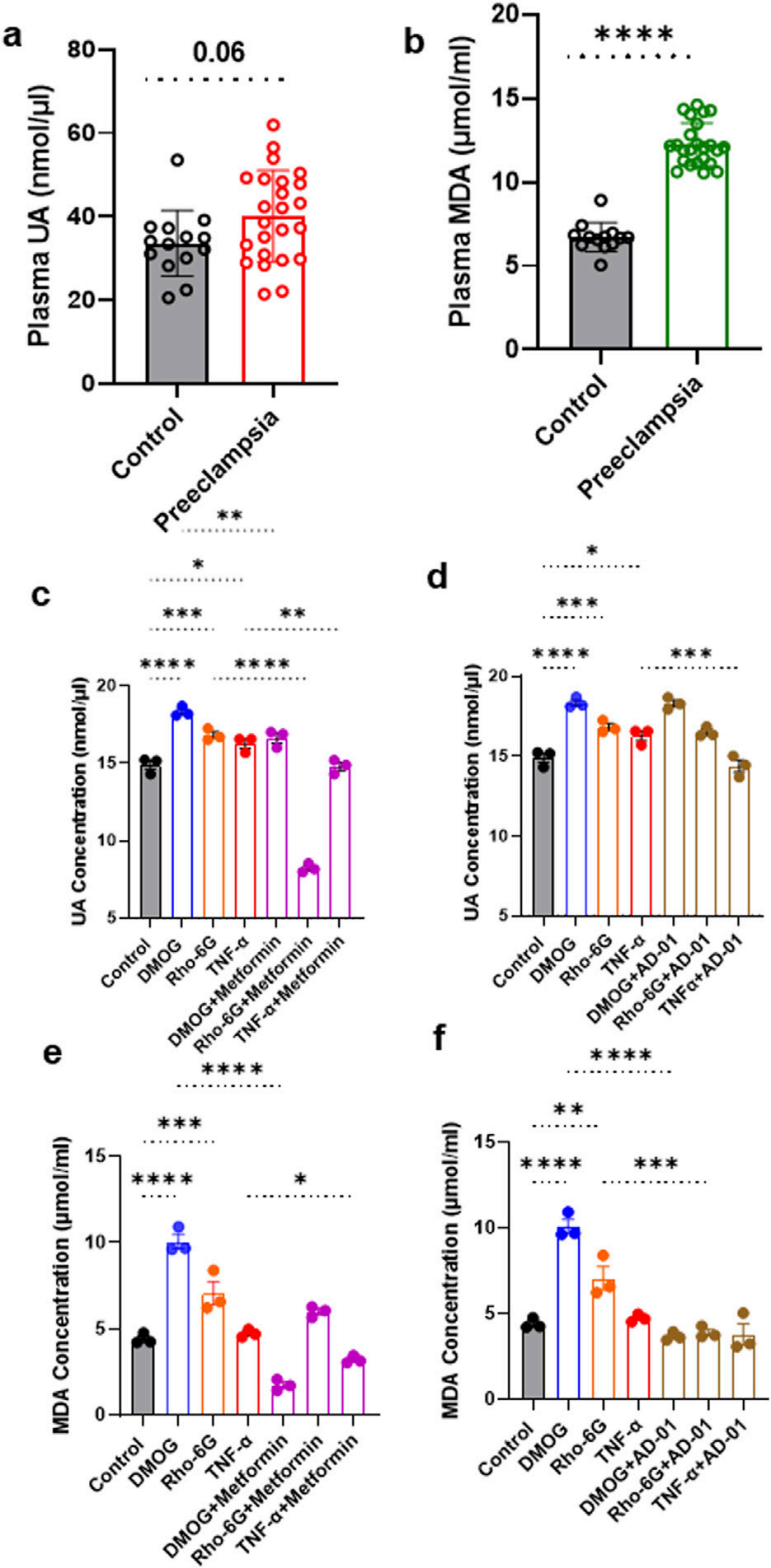
UA and MDA are increased in plasma from women with preeclampsia and cell lysates from 2D first trimester trophoblast *in vitro* models of preeclampsia. **(A, B)** UA and MDA concentrations were measured in plasma samples from individuals with preeclampsia or normotensive controls. Absorbances were recorded at 570 nm. **(C)** Metformin or **(D)** AD-01 treatment abrogated the increase in UA and MDA under certain preeclampsia-like conditions. ACH-3P cells were exposed to (DMOG, 1 mM) or (Rho-6G, 1 μg/mL) or (TNF-α, 10 ng/mL) to mimic hypoxia or mitochondrial dysfunction or inflammatory condition, respectively, and treated with metformin (0.5 mM), or AD-01 (100 nM), for 48 h. Untreated cells were used as controls. **(C, D)** UA and **(E, F)** MDA concentration was measured in ACH-3P cell lysates following the addition of treatments. **(A, B)** The data was plotted as mean ± SD; n ≥ 13; unpaired student’s t test; **(C–F)** The data was analyzed by one-way analysis of variance (ANOVA) with Sidak’s post-hoc test; and expressed as mean ± SEM; n = 3; *p < 0.05, **p < 0.01, ***p < 0.001, ****p < 0.0001.

Given UA and MDA are the most reliable oxidative stress biomarkers in preeclampsia that are detectable in plasma and highly secreted by placental cells (Afrose et al., 2022; Khaliq et al., 2018; Rani et al., 2010; Masoura et al., 2015), we quantified these biomarkers in both patient samples and *in vitro* trophoblast models of preeclamptic placenta to validate the model. Based on the clinical characteristics of our patient cohort, as expected, blood pressure was higher and gestational age at delivery lower in the preeclampsia group compared to normotensive controls. The groups were matched for maternal age, BMI and blood glucose levels (Table 1). UA concentrations showed a trend towards an increase in individuals with preeclampsia compared to healthy controls (Control 33.55 ± 7.87 nmol/μL vs. Preeclampsia 40.13 ± 10.88 nmol/μL, p = 0.06; Figure 1A; Table 1).

Although no significant correlation was observed between plasma UA and preeclampsia (r = 0.315, p = 0.06, Table 2), this became statistically significant after adjusting for gestational age as a confounding factor (r = 0.366, p = 0.03, Table 2).

**TABLE 2:** Adjusted correlations for differences in gestational age between plasma samples from pregnant women with preeclampsia or normotensive pregnancies for UA.

Plasma samples	UA	
	Pearson correlation	Correlation adjusted for GA
PE	r = 0.315	r = 0.366
	P = 0.061	*p = 0.030

Bold and * indicates statistical significance (p < 0.05). Key: UA, Uric acid; PE, preeclampsia; GA, gestational age.

Plasma MDA concentration was significantly increased in the individuals with preeclampsia compared to healthy controls (Control 6.72 ± 0.86 μmol/mL vs. Preeclampsia 12.27 ± 1.28 μmol/mL, p < 0.0001; Figure 1B; Table 3). Furthermore, there was a significant positive correlation between plasma MDA and preeclampsia (r = 0.924, p < 0.001), even when adjusted for differences in gestational age (r = 0.867, p < 0.001; Table 3).

**TABLE 3:** Adjusted correlations for differences in gestational age between plasma samples from pregnant women with preeclampsia or normotensive pregnancies for MDA.

Plasma samples	MDA	
	Pearson correlation	Correlation adjusted for GA
PE	r = 0.924	r = 0.867
	**p < 0.001	**p < 0.001

Bold and ** indicates statistical significance (p < 0.001). Key: MDA, Malondialdehyde; PE, preeclampsia; GA, gestational age.

In 2D *in vitro* trophoblast cell models of preeclampsia, UA was increased in response to all three stimuli (hypoxia mimetic, mitochondrial dysfunction, and inflammation) in comparison to control, at 48 h (Control 14.89 ± 0.30 nmol/μL vs. DMOG 18.32 ± 0.19 nmol/μL vs. Rho-6G 16.82 ± 0.22 nmol/μL vs. TNF-α 16.26 ± 0.29 nmol/μL, p < 0.0001; Figure 1C). Here, we tested only metformin and AD-01, given resveratrol is a well-known antioxidant agent (Zou et al., 2014; Lacerda et al., 2023) and aspirin is already used clinically for prevention of preeclampsia and has demonstrated anti-oxidant properties (Rolnik et al., 2017; Bujold et al., 2010; Duley et al., 2007). This increase in UA concentration was abrogated by metformin (0.5 mM) treatment in response to all three stimuli (DMOG 18.32 ± 0.19 nmol/μL vs. DMOG + Metformin 16.63 ± 0.31 nmol/μL, p < 0.01; Rho-6G 16.82 ± 0.22 nmol/μL vs. Rho-6G + Metformin 8.23 ± 0.16 nmol/μL, p < 0.0001; TNF-α 16.26 ± 0.29 nmol/μL vs. TNF-α+Metformin 14.79 ± 0.26 nmol/μL, p < 0.01; Figure 1C). However, only inflammation-induced higher UA concentration was abrogated by AD-01 (100 mM) treatment (TNF-α 16.26 ± 0.29 nmol/μL vs. TNF-α+AD-01 14.37 ± 0.38 nmol/μL, p < 0.001; Figure 1D).

Similarly, a significant increase in MDA concentration was demonstrated in ACH-3P cell lysate in response to hypoxia mimetic and mitochondrial dysfunctional stimuli in comparison to control (Control 4.41 ± 0.18 μmol/mL vs. DMOG 10.07 ± 0.42 μmol/mL vs. Rho-6G 7.06 ± 0.67 μmol/mL, p < 0.0001; Figure 1E) but inflammation did not have any effect on MDA cell concentration (Control 4.41 ± 0.18 μmol/mL vs. TNF-α 4.73 ± 0.13 μmol/mL, p = 0.985; Figure 1E). Higher MDA concentration in the presence of hypoxia mimetic was abrogated by metformin treatment (DMOG 10.07 ± 0.42 μmol/mL vs. DMOG + Metformin 1.72 ± 0.19 μmol/mL, p < 0.0001; Figure 1E). Surprisingly, even though MDA concentration was not significantly increased by inflammatory stimuli (TNF-α) compared to control, it was reduced when metformin treatment was added (TNF-α 4.72 ± 0.13 μmol/mL vs. TNF-α+Metformin 3.20 ± 0.12 μmol/mL, p < 0.05; Figure 1E). We also found that mitochondrial dysfunction induced by Rho-6G led to an increase in MDA concentration (p < 0.001), which was not abrogated by metformin treatment (p > 0.05; Figure 1E). The increase in MDA concentration driven by hypoxia mimetic or mitochondrial dysfunction was abrogated by AD-01 (100 nM) treatment at 48 h (DMOG 10.07 ± 0.42 μmol/mL vs. DMOG + AD-01 3.66 ± 0.14 μmol/mL, p < 0.0001; Rho-6G 7.06 ± 0.67 μmol/mL vs. Rho-6G + AD-01 3.89 ± 0.19 μmol/mL, p < 0.001; Figure 1F), unlike inflammation-induced oxidative stress (p > 0.05; Figure 1F). This suggests a differential mechanism for AD-01 in the presence of different types of placental stresses.

### Trophoblast proliferation is restored by metformin, aspirin or AD-01 treatment in the presence of hypoxia mimetic or oxidative stress, whereas resveratrol only restores hypoxia-induced inhibition of cell proliferation

**FIGURE 2:**
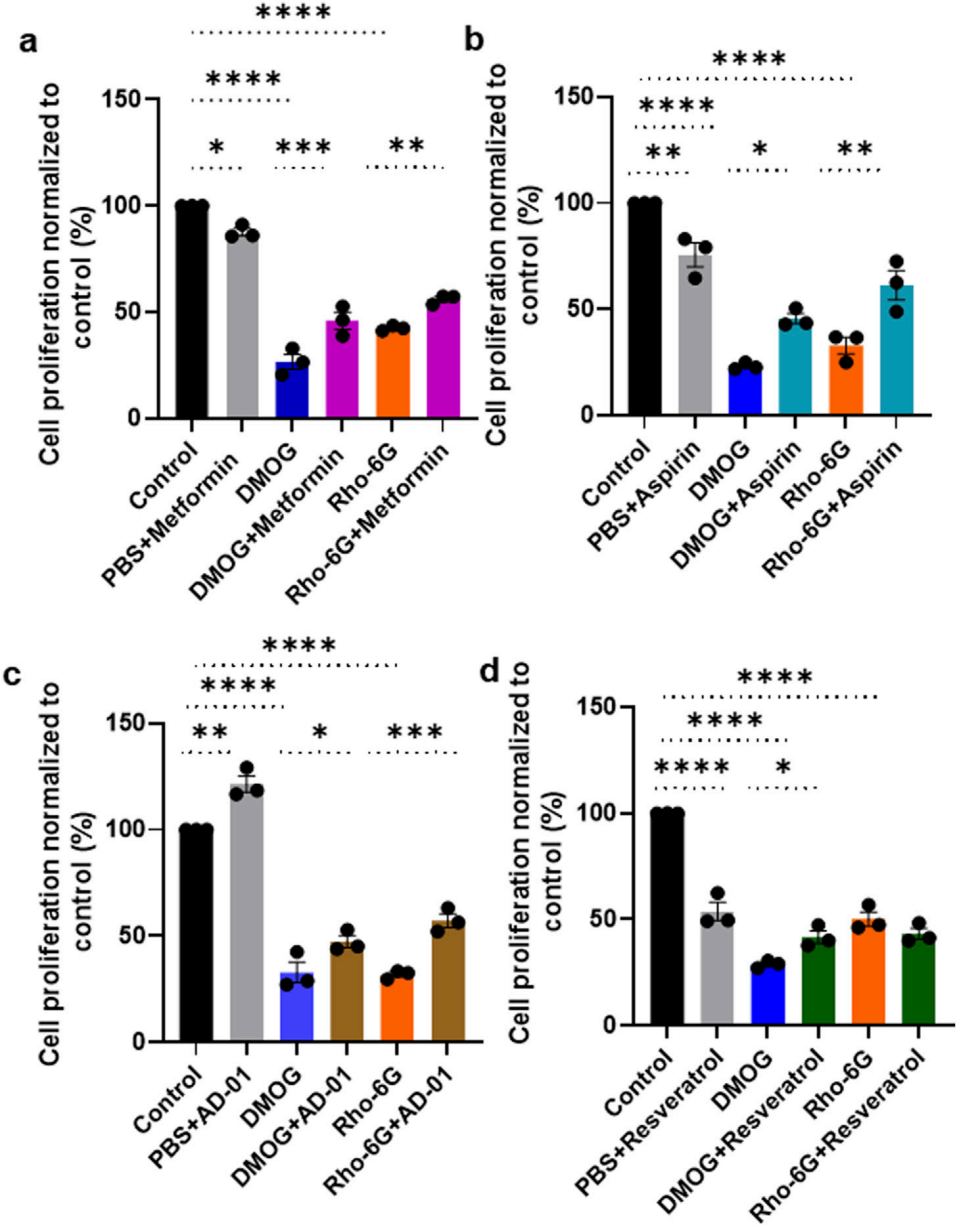
Metformin, aspirin and AD-01 improve cell proliferation in the presence of hypoxia and oxidative stress, whereas resveratrol only improves cell proliferation in hypoxic conditions. ACH-3P cells were treated with DMOG (1 mM) or Rho-6G (1 μg/mL) to emulate hypoxia or oxidative stress, respectively, ± **(A–D)** PBS ± **(A)** metformin (0.5 mM), or **(B)** aspirin (0.5 mM), or **(C)** AD-01 (100 nM) or **(D)** resveratrol (15 µM) for 48 h. MTT assay was performed as per manufacturer’s instructions and absorbance recorded at 565 nm. Data was analyzed by one-way ANOVA with Sidak’s post-hoc test; and expressed as mean ± SEM; n = 3; *p < 0.05; **p < 0.01; **p < 0.001; ****p < 0.0001.

Trophoblast proliferation in early gestation appears to be impaired in pregnancies that proceed to develop preeclampsia (Farah et al., 2020). Here, we investigated the ability of metformin, aspirin, AD-01 and resveratrol to restore proliferation or viability of trophoblast cells (ACH-3Ps) under hypoxia-like (DMOG) and mitochondrial dysfunction (Rho-6G) stresses (Figures 2A–D). As part of optimization, we conducted a time-course to include 24 h, 48 h and 72 h long treatments with preeclampsia-like stimuli ± treatments (Supplementary Figures S1–S3). We determined that 48 h-long treatments produced the most suitable response and were taken forward (Figure 2). Indeed, both DMOG and Rho-6G reduced the metabolic activity and proliferation of ACH-3Ps cells by ∼ 70% (p < 0.0001) and ∼ 60% (p < 0.0001), compared to the control, respectively. Metformin, aspirin, AD-01 and resveratrol rescued hypoxia-induced cell damage (DMOG 26.63% ± 3.58% vs. DMOG + Metformin 45.92% ± 4.02%, p < 0.001; Figure 2A), (DMOG 23.25% ± 0.93% vs. DMOG + Aspirin 45.70% ± 2.41%, p < 0.01; Figure 2B), (DMOG 32.91% ± 4.87% vs. DMOG + AD-01 47.40% ± 2.81%, p < 0.05; Figure 2C), and (DMOG 28.90% ± 1.02% vs. DMOG + Resveratrol 41.67% ± 2.93%, p < 0.05; Figure 2D). Similarly, metformin, aspirin and AD-01 restored cell proliferation following induction of mitochondrial dysfunction (Rho-6G 42.44% ± 0.74% vs. Rho-6G + Metformin 56.11% ± 1.30%, p < 0.01; Figure 2A), (Rho-6G 32.94% ± 3.91% vs. Rho-6G + Aspirin 61.34% ± 6.85%, p < 0.01; Figure 2B), (Rho-6G 31.97% ± 1.16% vs. Rho-6G + AD-01 57.28% ± 3.20%, p < 0.001; Figure 2C), whereas resveratrol was not able to rescue ACH-3Ps cell proliferation in these preeclampsia-like conditions (Rho-6G 50.16% ± 3.33% vs. Rho-6G + Resveratrol 43.22% ± 2.57%, p > 0.05; Figure 2D). Interestingly, whilst aspirin (p < 0.01; Figure 2B) and metformin (p < 0.05; Figure 2A) seem to modestly impair trophoblast proliferation under physiological conditions (PBS), AD-01 did not have this effect but shows improved cell proliferation (p < 0.01; Figure 2C).

### Hypoxia mimetic inhibits trophoblast cell migration while metformin, aspirin, and AD-01 restore it

**FIGURE 3:**
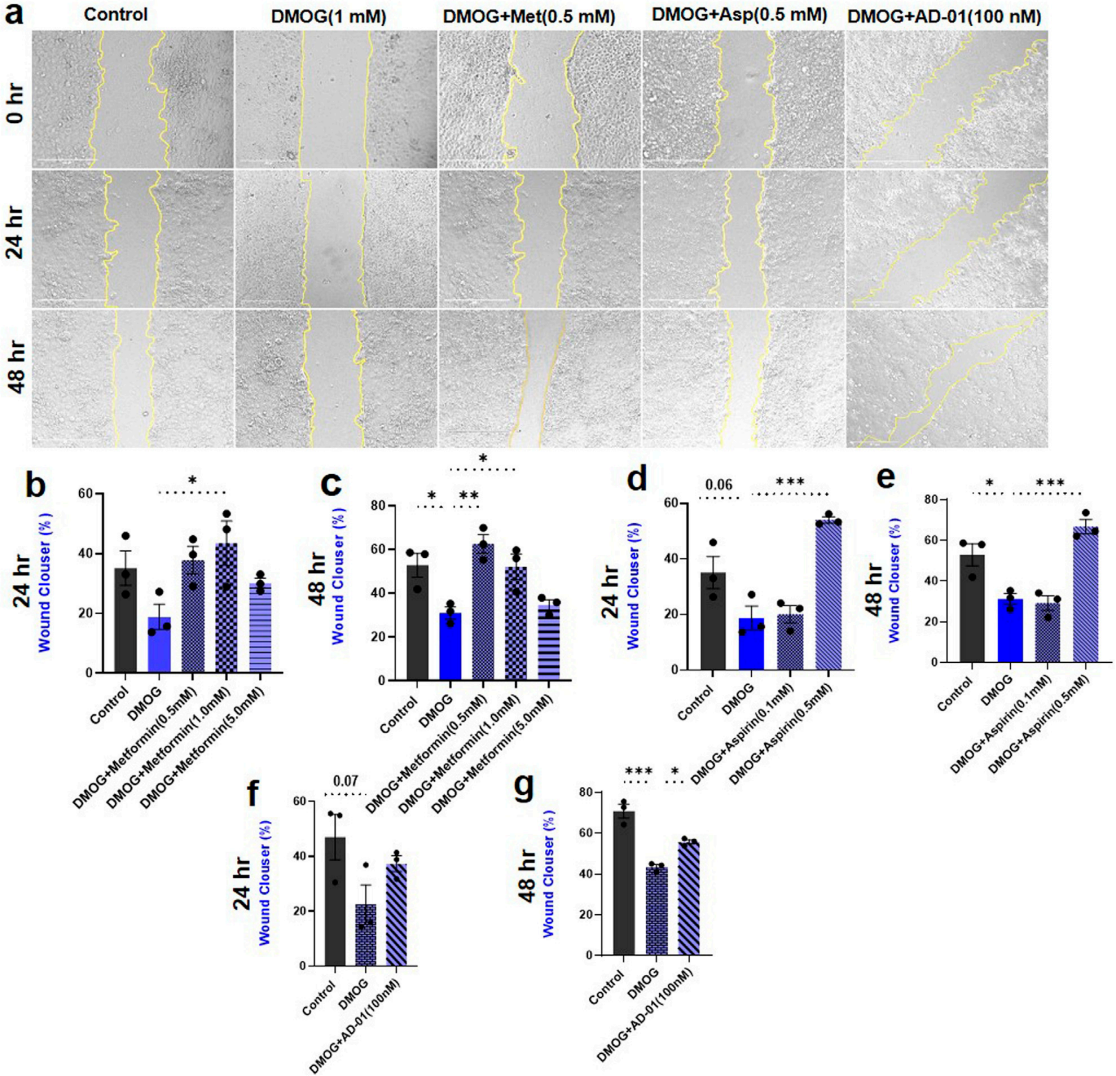
Hypoxia reduced trophoblast cell migration only at 48 h time point, and metformin, aspirin, AD-01 restore it to normal. **(A)** Representative images of the wound scratch assay with ACH-3P cells, following treatment with DMOG (1 mM) to mimic hypoxia, and metformin (Met) (0.5 mM), aspirin (asp) (0.5 mM), or AD-01 concentration (100 nM) at t = 0 h, t = 24 h and t = 48 h after wound scratch. Untreated cells were used as controls. The scale bar indicates 400 µm. **(B, C)** Percentage area of wound closure from 0 h to 24 h and 0 h to 48 h following treatment with various metformin doses (0.5 mM, 1 mM and 5 mM) under hypoxia. **(D, E).** Percentage area of wound closure from 0 h to 24 h and 0 h to 48 h following treatment with two different doses of aspirin (0.5 mM, 0.1 mM) under hypoxia. **(F, G)** Percentage area of wound closure from 0 h to 24 h and 0 h to 48 h following treatment with AD-01 (100 nM) under hypoxia. The data is analyzed by one-way ANOVA with Sidak’s post hoc test and expressed as mean ± SEM; n = 3; ***p < 0.05, **p < 0.01, ***p < 0.001.

The root cause of preeclampsia is aberrant placentation, which often involves impaired migration of trophoblasts and remodeling of SUA, in the presence of extended periods of hypoxia (McNally et al., 2017). Therefore, to assess the impact of pathological hypoxia, and evaluate potential treatments (metformin, aspirin and AD-01) for preeclampsia prevention, a wound scratch assay was conducted. The effect of resveratrol was not tested here because of its high toxicity on trophoblast proliferation/survival and inconsistent restorative cell proliferation effects in DMOG- or Rho-G-treated trophoblasts (Figure 2D). Trophoblast cell migration was quantified using ImageJ software in order to calculate the percentage wound closure following 24 h or 48 h treatment (Figure 3A). There was no significant reduction in trophoblast cell migration as a result of HIF-1α activation by DMOG treatment at 24 h although there was a downward trend (Figures 3A, B). Metformin at one dose only (1.0 mM) was able to improve trophoblast migration compared to hypoxia/DMOG at 24 h time point (DMOG 18.87% ± 4.22% vs. DMOG + Metformin (1.0 mM) 43.46% ± 7.46%, p < 0.05; Figure 3B). However, at 48 h, DMOG treatment induced statistically significant reduction in trophoblast migration (Control 52.81% ± 5.47% vs. DMOG 31.09% ± 2.66%, p < 0.05; Figure 3C), which was restored by metformin treatment back to the control levels at two different concentrations (0.5 mM and 1.0 mM) (DMOG 31.09% ± 2.66%, vs. DMOG + Metformin (0.5 mM) 62.58% ± 4.25%, p < 0.01; vs. DMOG ± Metformin (1.0 mM) 52.14% ± 5.83%, p < 0.05; Figure 3C). At both time points, aspirin had the same effect only at one concentration (0.5 mM) that improved trophoblast migration at 24 h (DMOG 18.87% ± 4.22% vs. DMOG + Aspirin 54.05% ± 1.11%, p < 0.001; Figure 3D) and 48 h (DMOG 31.09% ± 2.66% vs. DMOG + Aspirin 66.67% ± 3.51%, p < 0.001; Figure 3E), compared to DMOG.

Given that metformin and aspirin have already been tested for the treatment or prevention of preeclampsia in humans, respectively (Cluver et al., 2021; Duley et al., 2019), we also wanted to explore novel treatments including FKBPL-based peptide mimetic, AD-01. Following 24 h treatment with DMOG, a similar trend in reduction of trophoblast cell migration was observed, which was not restored by AD-01 at this time point (Control 46.93% ± 8.24% vs. DMOG 22.34% ± 7.25%, p = 0.07; Figure 3F). However, there was statistically significant reduction in trophoblast migration induced by hypoxia at 48 h (Control 70.84% ± 3.49% vs. DMOG 43.39% ± 1.24%, p < 0.001; Figure 3G), which was improved with AD-01 treatment (100 nM) (DMOG 43.39% ± 1.24% vs. DMOG + AD-01 55.72% ± 1.03%, p < 0.05; Figure 3G).

### Mitochondrial dysfunction increases pro-inflammatory cytokine levels, which could be potentially abrogated by metformin

**FIGURE 4:**
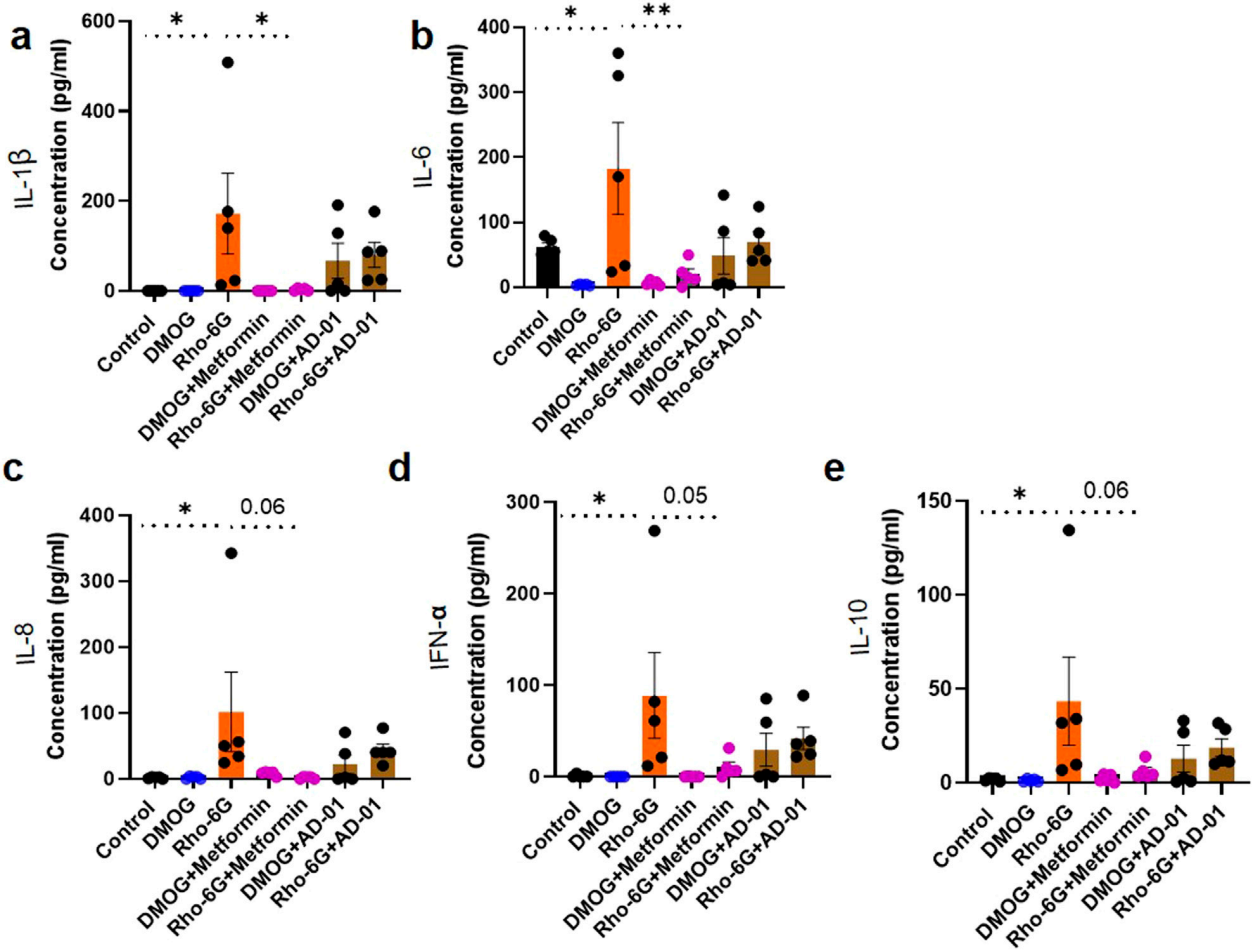
Pro-inflammatory cytokines were increased following induction of mitochondrial dysfunction and metformin showed some potential at abrogating this increase in the first trimester trophoblast cells. **(A–E)** ACH-3P cells were treated with DMOG (1 mM) or Rho-6G (1 μg/mL) to mimic hypoxia, or mitochondrial dysfunction, respectively, ± metformin (0.5 mM), or AD-01 (100 nM), for 48 h. Cell supernatants were collected to determine **(A–D)** the pro-inflammatory (IL-1β, IL-6, IL-8, IFN-α) and **(E)** anti-inflammatory cytokine (IL-10) concentrations by Cytometric Bead Array. Untreated cells were used as control groups. Pro-inflammatory cytokines **(A)** IL-1β, **(B)** IL-6, **(C)** IL-8, **(D)** IFN-α levels were increased following mitochondrial dysfunction induction and IL-1β and IL-6 increase was abrogated by metformin treatment in ACH-3Ps. **(E)** Anti-inflammatory IL-10 cytokine was also increased in the presence of mitochondrial dysfunction, which was borderline reduced with metformin. The data was analyzed by one-way analysis of variance (ANOVA) with Sidak’s post-hoc test and expressed as mean ± SEM; n = 5; *p < 0.05; **p < 0.01.

The imbalance between pro-inflammatory and anti-inflammatory cytokines in preeclampsia promotes inflammation and oxidative stress, contributing to the development of endothelial dysfunction, hypertension, and potentially organ damage. (Martínez-Varea et al., 2014; Geldenhuys et al., 2018). In this inflammatory and oxidative stress environment, the anti-inflammatory cytokine, IL-10, fails to effectively counteract pro-inflammatory cytokines, which contributes to the progression of the disease (Szarka et al., 2010; Afshari et al., 2005; Udenze et al., 2015). In our *in vitro* first trimester trophoblast cell model of placental stress in preeclampsia, we wanted to determine the inflammatory mechanism of hypoxia mimetic or mitochondrial dysfunction with or without metformin or AD-01. Resveratrol was not included here due to its toxic effects on trophoblast proliferation demonstrated in Figure 2D and aspirin is a well-known anti-inflammatory agent. Pro-inflammatory cytokine concentrations of IL-1β (Control 0.00 ± 0.00 pg/mL vs. Rho-6G 172.3 ± 89.92 pg/mL, p < 0.05, Figure 4A), IL-6 (Control 62.66 ± 5.59 pg/mL vs. Rho-6G 182.7 ± 70.63 pg/mL, p < 0.05, Figure 4B), IL-8 (Control 1.99 ± 0.70 pg/mL vs. Rho-6G 101.8 ± 60.49 pg/mL, p < 0.05, Figure 4C), IFN-α (Control 0.74 ± 0.74 pg/mL vs. Rho-6G 88.98 ± 46.76 pg/mL, p < 0.05, Figure 4D) were increased significantly following treatment with mitochondrial dysfunction agent, Rho-6G. Metformin showed some potential at abrogating the increase in IL-1β (Rho-6G 172.3 ± 89.92 pg/mL vs. Rho-6G + Metformin 2.93 ± 1.28 pg/mL, p < 0.05, Figure 4A) and IL-6 (Rho-6G 182.7 ± 70.63 pg/mL vs. Rho-6G + Metformin 20.09 ± 8.34 pg/mL, p < 0.01, Figure 4B). Borderline significance was observed in relation to the metformin-mediated reduction of IL-8 (Rho-6G 101.8 ± 60.49 pg/mL vs. Rho-6G + Metformin 8.57 ± 1.51 pg/mL, p = 0.06, Figure 4C) and IFN-α (Rho-6G 88.98 ± 46.76 pg/mL vs. Rho-6G + Metformin 10.70 ± 5.38 pg/mL, p = 0.05, Figure 4D) in the presence of mitochondrial dysfunction. On the other hand, anti-inflammatory cytokine, IL-10 was also increased as a result of mitochondrial dysfunction and metformin (0.5 mM) treatment borderline normalized its concentration (Control 1.80 ± 0.34 pg/mL vs. Rho-6G 43.44 ± 23.46 pg/mL, p < 0.05; Rho-6G 43.44 ± 23.46 pg/mL vs. Rho-6G + Metformin 6.32 ± 1.97 pg/mL , p = 0.06, Figure 4E).

In this experiment, although AD-01 showed a trend towards a reduction in pro-inflammatory cytokines (IL-1β, IL-6, IL-8, IFN-α) and IL-10, this was not statistically significant. Interestingly, pro-inflammatory cytokines were not increased in DMOG/hypoxic condition.

## Discussion

Preeclampsia is an understudied cardiovascular disorder of pregnancy, however despite some progress, no definitive treatment options currently exist. Several factors have contributed to this, including difficulties in obtaining human samples of the early placenta and the lack of biologically relevant model systems of this disease. In order to develop better monitoring and preventative strategies for pregnant individuals and their offspring at risk of or affected by preeclampsia, reliable and representative models of the early placenta are necessary that recapitulate multifactorial nature of the disease (Ghorbanpour et al., 2023). The root cause of preeclampsia is inappropriate placentation, often related to oxidative stress, aberrant angiogenesis, and/or inflammation (Burton et al., 2019). Our recent meta-analysis based on nine clinical studies identified the three most reliable oxidative stress-related diagnostic biomarkers for preeclampsia including Ischemia-Modified Albumin (IMA), UA, and MDA (Afrose et al., 2022). In this study, we confirmed the increased concentration of UA and MDA in human plasma samples from individuals with preeclampsia, compared to healthy controls. Then, we developed *in vitro* first trimester trophoblast cell models of placental stress preceding preeclampsia that are reflective of three different aspects of its pathogenesis: hypoxia, inflammation and mitochondrial dysfunction (Palei et al., 2013). Using these models, we tested a number of clinically available and novel therapeutics for preeclampsia including aspirin, metformin, resveratrol and FKBPL-based therapeutic peptide mimetic, AD-01 (Cluver et al., 2019; Bujold et al., 2010; Caldeira-Dias et al., 2019; Ghorbanpour et al., 2024). We showed that (i) metformin, and AD-01 can abrogate upregulation of UA and MDA due to preeclampsia-like placental stresses, (ii) metformin, aspirin and AD-01 can improve impaired cell proliferation and migration due to hypoxia-like and mitochondrial dysfunction placental stresses, and (iii) metformin mitigates certain aspects of heightened inflammation as a result of mitochondrial dysfunction. Overall, metformin showed the most promising therapeutic effect across all types of placental stresses and assays. Surprisingly, based on our proliferation or viability assay, all drugs (aspirin, metformin and resveratrol) except AD-01 in physiological conditions had a negative impact on trophoblast proliferation measured by the metabolic activity, suggesting a good safety profile of AD-01, which needs to be investigated further in *in vivo* studies. This was an unexpected result for metformin, aspirin and resveratrol, which are clinically utilized or trialled for preeclampsia prevention/treatment and are deemed safe to use in pregnancy. Nevertheless, their mechanism of action related to trophoblast cells specifically is still not well-understood, which this study elucidated further. Our findings suggest that the inhibitory effects of aspirin, metformin, or resveratrol on trophoblast proliferation might be context-dependent, reflecting their ability to modulate stress-induced cellular responses rather than direct cytotoxicity.

Here we introduce, for the first time, a simple cell model mimicking specific placental stresses preceding preeclampsia using a custom-made first trimester trophoblast cell line that closely resembles primary trophoblasts (Hiden et al., 2007). In our *in vitro* models, preeclampsia-related stresses including hypoxia, inflammation and mitochondria dysfunction were individually mimicked, enabling the evaluation of therapeutic potential of treatments for each aspect of the disease’s pathogenesis, paving the way for personalized medicine in preeclampsia. Nevertheless, in the future, various combinations of these placental stresses can be used concurrently.

In this study, we measured UA and MDA oxidative stress markers intracellularly in ACH-3P trophoblast cells, given their strong association with preeclampsia as demonstrated in our previously published meta-analysis, and their role in the pathogenesis of preeclampsia (Afrose et al., 2022; Masoura et al., 2015; Khaliq et al., 2018; Yoneyama et al., 2002; Rani et al., 2010). UA is produced in the liver through purine metabolism and nutritional sources. Preeclampsia is directly associated with placental ischemia and oxidative stress, which can trigger or activate the release of xanthine oxidase enzyme (Myatt and Cui, 2004). UA is formed when adenosine triphosphate is cleaved into adenosine xanthine by xanthine oxidase (Uaa et al., 2017). Many studies have demonstrated that serum UA levels increase with preeclampsia severity, and that UA may play an important role in the pathogenesis of preeclampsia (Sudjai and Satho, 2022; Masoura et al., 2015; Khaliq et al., 2018). Here, we confirmed that following the diagnosis of preeclampsia, UA plasma concentration was increased albeit borderline significant, and this was also reflected in our 2D first trimester trophoblast cell- based *in vitro* models of placental stress in preeclampsia. Significant positive correlation was demonstrated between UA and preeclampsia when adjusted for differences in gestational age at sample collection. In our trophoblast *in vitro* models, UA was increased as a result of all stress stimuli (hypoxic mimetic, inflammation and mitochondrial dysfunction).

More prominent than UA, plasma MDA was significantly higher in preeclampsia compared to healthy controls. However, this was reflected in some of our *in vitro* models where hypoxia or mitochondrial dysfunction was induced, but not in the presence of inflammation. As a three-carbon aldehyde, MDA produces free radicals, which can damage cell membranes and are correlated with the severity of the disease (Sim et al., 2003). A common form of oxidative stress, called uncontrolled lipid peroxidation, can contribute to pregnancy complications, including preeclampsia (Rani et al., 2010; Ferreira et al., 2020). Numerous previous studies have indicated that MDA is elevated in various diseases, including pregnancy-induced hypertension and preeclampsia (Rani et al., 2010; Adiga et al., 2007; Ferreira et al., 2020). Given that in preeclampsia, there is an imbalance between free radicals and antioxidants that results in oxidative stress and, consequently, increased levels of lipid peroxide (Rani et al., 2010), MDA as a biomarker and a therapeutic target shows promise towards clinical translation.

Even though primary human cytotrophoblasts or trophoblast stem cells are the gold standard as *in vitro* placental models, these cells undergo rapid changes once they are cultured, are highly variable between individuals, and do not proliferate, hence have limited utility for drug screening. Although immortalized cell lines are generally considered less representative of the human placenta, they are readily available and can be expanded to meet the requirements of large-scale applications with good reproducibility (Zhao et al., 2021). Previous work has shown that ACH-3Ps contain both cytotrophoblasts that express integrin α6β4 and HLA-G-positive extravillous trophoblasts (EVTs) expressing integrin α5β1, and matrix metalloproteinases, MMP2 and MMP9, unlike other cell lines including HTR-8/SVneo (Hiden et al., 2007). While ACH-3Ps are not able to undergo syncytialization in 2D culture, the syncytiotrophoblast marker, β-human chorionic gonadotropin (β-hCG), was detectable using pregnancy test in our ACH-3Ps cell culture *in vitro.* The karyotyping of this cell line revealed a male gender, and transcriptomic analysis confirmed a close alignment between the gene expression profiles of ACH-3Ps and their primary trophoblast cell origin. In a microfluidic device, ACH-3P cells were shown to contain both HLA-G- and EpCAM- positive extravillous and villous first trimester trophoblast subpopulation, respectively, which are vital for the development of the placenta (Ghorbanpour et al., 2023). ACH-3Ps are also highly proliferative, enabling large-scale production, which was necessary for quantifying UA and MDA in cell lysates. Therefore, we were able to generate 2D *in vitro* models of placental stress often observed in preeclampsia that were reproducible, low-risk, and low-cost by using this cell line.

Although the pathogenesis of preeclampsia is still poorly understood, the root cause is inappropriate remodelling of the SUA, likely caused by inadequate trophoblast cell function. Subsequently, chronic placental hypoxia follows, leading to restrictive supply of oxygen and nutrients to the fetus (McNally et al., 2017). This can lead to inflammation, mitochondrial dysfunction and oxidative stress within the placental cells, particularly trophoblast cells. Mitochondrial dysfunction in preeclampsia or other obstetrics complications such as gestational diabetes mellitus can lead to impaired energy production and an increased generation of ROS, which in turn leads to endothelial dysfunction, another hallmark feature of preeclampsia (Ferreira et al., 2020; Fisher et al., 2021). In fact, mitochondrial dysfunction is a major source of intracellular and extracellular oxidative stress, as demonstrated within placental tissues from women with preeclampsia (Han et al., 2020; Lian et al., 2022).

In our *in vitro* first trimester trophoblast model of placental stress, we induced hypoxia chemically by DMOG, an activator of HIF-1α and an inhibitor of prolyl-hydroxylases (Nevo et al., 2006; Zippusch et al., 2021; Sasagawa et al., 2021; Zhao et al., 2021). Mitochondrial dysfunction leading to oxidative stress was induced by Rho-6G, a lipophilic dye and a potent inhibitor of oxidative phosphorylation (Dutra Silva et al., 2021; Gear, 1974). TNF-α is a key proinflammatory cytokine involved in preeclampsia pathogenesis (Ghorbanpour et al., 2023), and its effects on trophoblast cells are directly relevant to understanding the interplay between inflammation and oxidative stress in this condition. Inflammation is a key driver of oxidative stress in preeclampsia, as highlighted in our recent review (Afrose et al., 2025), where we discussed how inflammatory processes exacerbate reactive oxygen species (ROS) production, contributing to the oxidative stress observed in this condition. We used these cell-based models to test a number of potential therapeutics for preeclampsia including (i) a well-established preventative treatment for preterm preeclampsia, aspirin (ACOG, 2019), (ii) a hypoglycaemic agent, metformin, emerging as a promising treatment of preeclampsia (Alqudah et al., 2018; Cluver et al., 2021; Decui et al., 2021), (iii) naturally occurring compound-derived drug, resveratrol (Shi et al., 2023), and (iv) a newly developed compound, AD-01, which is a based on an anti-angiogenic protein, FKBPL (McClements et al., 2013). These drugs are known to have pleiotropic effects, including anti-inflammatory, antioxidant, and/or angiogenesis-related properties, which are relevant to preeclampsia. The dosages of the drugs used in this study were representative of the plasma levels following drug absorption, metabolism, and distribution in humans (Angiolillo et al., 2022; Sheleme, 2021; LaMoia and Shulman, 2021; Seidler et al., 2018; Kemper et al., 2022; Singh et al., 2019; Arif and Aggarwal, 2023; Mather et al., 2021). Based on the cell proliferation or viability assay results, metformin, aspirin, resveratrol, or AD-01 may be useful for prevention of preeclampsia by reversing HIF-1α or oxidative stress-induced first trimester trophoblast cell damage. We found a significant reduction in trophoblast cell migration caused by hypoxia mimetic at 48 h.

Immune dysregulation is another major aspect of preeclampsia pathogenesis. The imbalance of pro-inflammatory and anti-inflammatory factors plays a crucial role in preeclampsia development and progression (Rana et al., 2019; Bisson et al., 2023; Weel et al., 2016; Peixoto et al., 2016). We demonstrated, in this study, that pro-inflammatory cytokines (IL-1β, IL-6, IL-8, IFN-α) levels were increased by oxidative stress and abrogated by metformin treatment only. Surprisingly, hypoxia mimetic did not affect pro-inflammatory cytokines levels. We also observed opposite trends in anti-inflammatory cytokine, IL-10, concentration in these experiments. Previous research has shown that IL-10 is downregulated in preeclampsia (Murray et al., 2022; Michalczyk et al., 2020). The increased production of IL-10 could be interpreted as a compensatory mechanism that mitigates the heightened inflammatory response in trophoblast cells by hypoxia (Opichka et al., 2021; Aneman et al., 2020). Further studies are needed to uncover this interaction in the context of preeclampsia.

While the effects and mechanisms of both placental stresses in preeclampsia and treatments should also be investigated in other placental cell types, including endothelial and immune cells, our 2D *in vitro* trophoblast cell model demonstrates value in studying the complex multifactorial pathophysiology of preeclampsia. A key limitation of our study is the use of a single trophoblast cell line (ACH-3P), which may not fully capture the complexity of preeclampsia pathophysiology. In support of this study findings, our previous work demonstrated that AD-01 effectively restores endothelial function and integrity following DMOG treatment, by regulating FKBPL and HIF-1α expression (Ghorbanpour et al., 2024). Given that DMOG is extensively known as HIF-1α mimic, we did not measure HIF-1α expression in trophoblast cells, however we demonstrated increased expression of oxidative stress biomarkers, UA and MDA, which are the most relevant to preeclampsia, in DMOG-treated trophoblasts. Future studies should measure the concentrations of well-known biomarkers of angiogenic imbalance in preeclampsia, sFlt-1 and PlGF (Dimitriadis et al., 2023) to further understand the mechanisms of these potential therapeutics in the context of specific preeclampsia-like stresses in our first trimester trophoblast *in vitro* models. This *in vitro* model can be improved by using primary first trimester trophoblast cells to further our understanding of these specific pathological mechanisms and therapeutic approaches, in preeclampsia. Our *in vitro* models can be used for high-throughput screening of potential therapeutics for preeclampsia to develop personalized treatments and gain a deeper understanding of the pathogenesis of this complex disease.

## Conclusion

In this study, we developed a simple, low-cost and reproducible 2D *in vitro* model of placental stresses preceding preeclampsia using the custom-made immortalized first trimester trophoblast cell line, ACH-3Ps, which closely resembles primary trophoblasts and contains all three trophoblast subtypes includes cytotrophoblasts, EVTs and syncytiotrophoblasts. All of these trophoblast subtypes are crucial for placental development and growth. We developed this model to mimic the features of hypoxia, mitochondrial dysfunction and inflammation of the preeclamptic placenta. Additionally, we explored therapeutic strategies that can abrogate these pathogenic mechanisms and potentially prevent the development of preeclampsia. We showed that metformin, aspirin, resveratrol and AD-01 show promise as treatments for preeclampsia, capable of abrogating hypoxia-, inflammation- or mitochondrial dysfunction-induced first trimester trophoblast cell damage. Metformin appears to be the most effective across different placental stresses typical of preeclampsia and can improve trophoblast proliferation and migration, as well as reduce oxidative stress and inflammation. This is the first study to report the therapeutic potential of the FKBPL-based therapeutic peptide, AD-01, which appears to be less toxic compared to other treatments tested in physiological conditions, in terms of trophoblast proliferation. However, metformin, aspirin and resveratrol have been extensively tested *in vivo* and in human studies including pregnancy, whereas FKBPL-based therapeutic peptides (AD-01 and ALM201) have only been tested in the context of cancer (El Helali et al., 2022). Future studies should test AD-01 in preclinical pregnancy models to determine its safety and efficacy including the impact on fetal health.

## Data Availability

The original contributions presented in the study are included in the article/Supplementary Material, further inquiries can be directed to the corresponding author.

## Supplementary material

SUPPLEMENTARY FIGURE S1 Time course optimisation related to the effects of aspirin in the presence of preeclampsia-like stimuli on trophoblast cell proliferation. ACH-3Ps cells were treated with DMOG (1 mM), TNF-α (10 ng/mL) or Rho-6G (1 μg/mL) to emulate hypoxia, inflammation or oxidative stress, ± aspirin (0.5 mM) respectively for (A) 24 h or (B) 72 h. MTT assay was performed as per manufacturer’s instructions and absorbance recorded at 565 nm minus absorbance at 650 nm. Data analyzed by one-way ANOVA Šídák’s multiple comparisons test; and expressed as mean ± SEM; n = 3; ****p < 0.0001.

SUPPLEMENTARY FIGURE S2 Time course optimisation related to the effects of metformin in the presence of preeclampsia-like stimuli on trophoblast cell proliferation. ACH-3Ps cells were treated with DMOG (1 mM), TNF-α (10 ng/mL) or Rho-6G (1 μg/mL) to emulate hypoxia, inflammation or oxidative stress, ± metformin (0.5 mM) respectively for (A) 24 h or (B) 72 h. MTT assay was performed as per manufacturer’s instructions and absorbance recorded at 565 nm minus absorbance at 650 nm. Data analyzed by one-way ANOVA Šídák’s multiple comparisons test; and expressed as mean ± SEM; n = 3; *p < 0.05; **p < 0.01; **p < 0.001; ****p < 0.0001.

SUPPLEMENTARY FIGURE S3 Time course optimisation related to the effects of resveratrol in the presence of preeclampsia-like stimuli on trophoblast cell proliferation. ACH-3Ps cells were treated with DMOG (1 mM), TNF-α (10 ng/mL) or Rho-6G (1 μg/mL) to emulate hypoxia, inflammation or oxidative stress, ± resveratrol (10 μM) respectively for (A) 24 h or (B) 72 h. MTT assay was performed as per manufacturer’s instructions and absorbance recorded at 565 nm minus absorbance at 650 nm. Data analyzed by one-way ANOVA Šídák’s multiple comparisons test; and expressed as mean ± SEM; n = 3; *p < 0.05; **p < 0.01; **p < 0.001; ****p < 0.0001.
