# Supplementary figures and images for: Evaluating oxidative stress targeting treatments in in vitro models of placental stress relevant to preeclampsia
Source: Front Cell Dev Biol. 2025 Feb 28;13:1539496. doi: 10.3389/fcell.2025.1539496 (PMC11920713; doi:10.3389/fcell.2025.1539496)

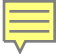

A

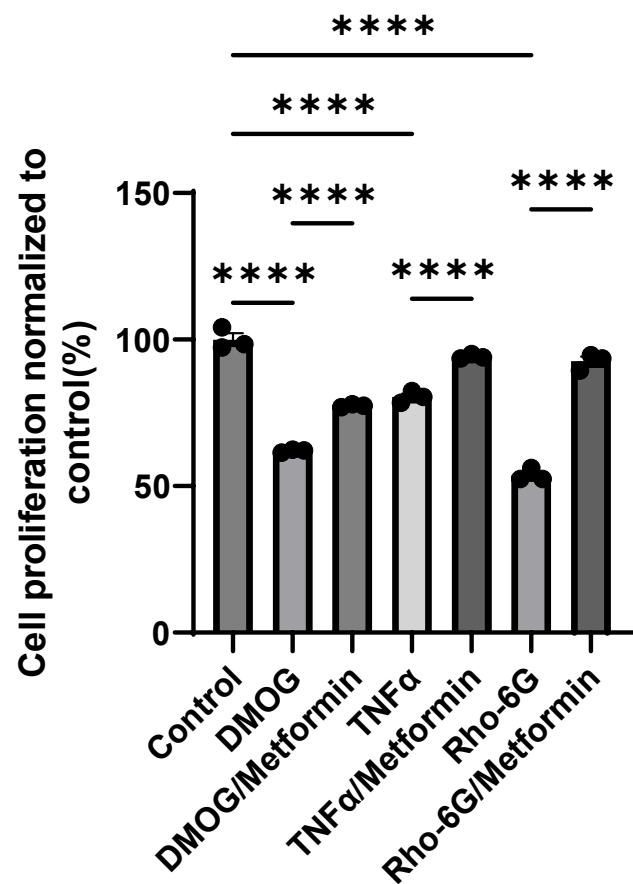

B

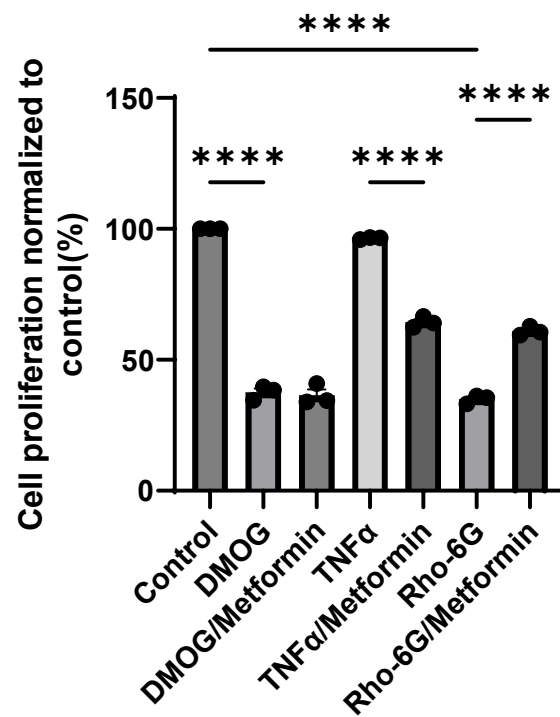

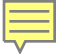

A

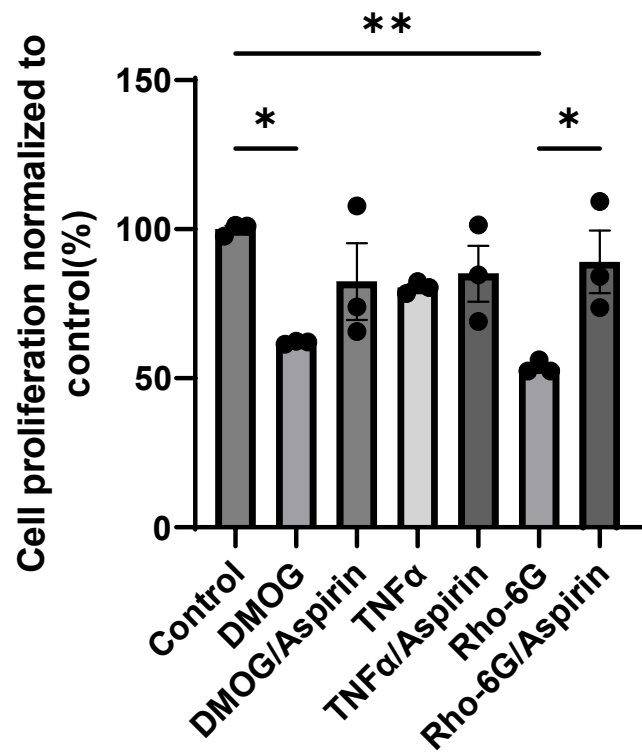

B

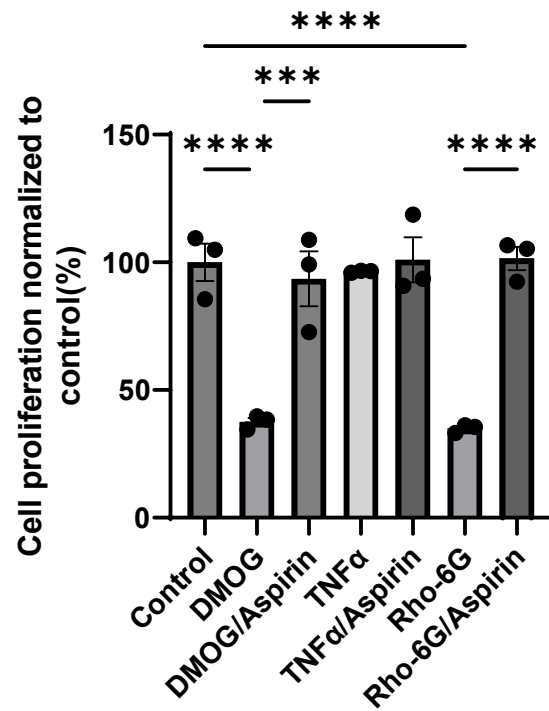

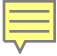

A

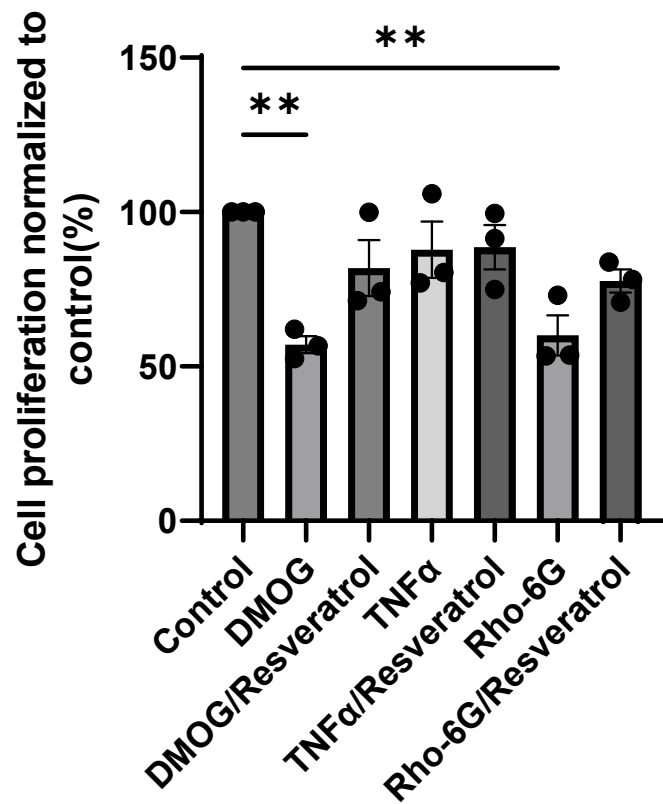

B

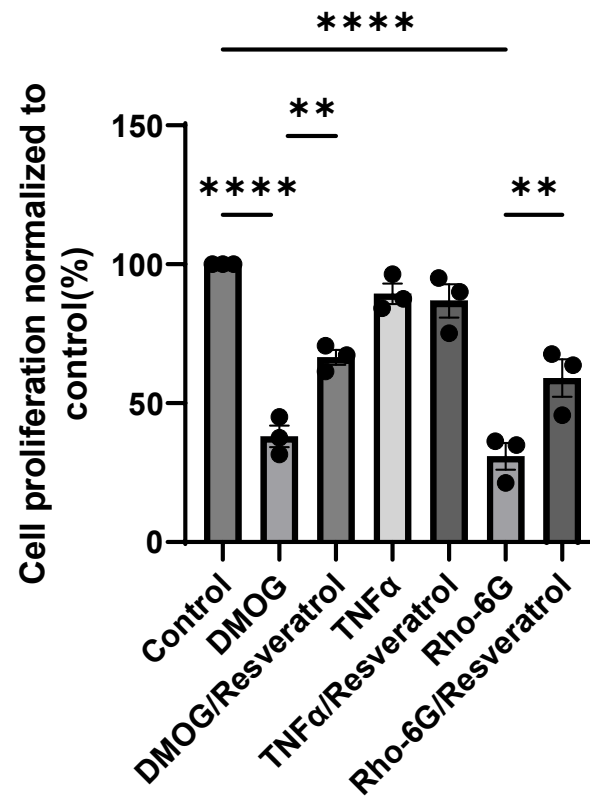

Supplement: Supplementary file 1 [file DataSheet1.pdf]
